# Livestock Dung Proxies Provide Insights into Grazing Density Quantification and Distribution

**DOI:** 10.3390/ani15192789

**Published:** 2025-09-25

**Authors:** Bo Huang, Yingying Liu, Yingxi Chen, Yixuan Dong, Fujiang Hou, Shenghua Chang, Shuhua Yi, Yi Sun

**Affiliations:** 1 State Key Laboratory of Herbage Improvement and Grassland Agro-Ecosystems, College of Pastoral Agriculture Science and Technology, Lanzhou University, Lanzhou 730000, China; huangbo22@mails.ucas.ac.cn (B.H.);; 2 School of Geographic Science, Nantong University, 9 Seyuan Road, Nantong 226007, China

**Keywords:** the Qinghai–Tibet plateau, household pasture, grazing intensity, unmanned aerial vehicle, alpine meadow

## Abstract

Managed grazing is the most widespread and economically significant form of grassland utilization worldwide. Accurate quantification of the spatiotemporal distribution of grazing intensity (GI) is crucial for promoting sustainable management of livestock-grassland ecosystems. However, a reliable method for dynamically monitoring GI and quantifying key proxies under real-world grazing conditions is still lacking. In this study, we developed a practical approach to estimate GI using sequential unmanned aerial vehicle (UAV) monitoring and evaluated its feasibility in a typical household pasture on the Qinghai–Tibetan Plateau, China. Our findings show that: (1) yak dung is clearly identifiable in UAV image, although detection accuracy decreases with increasing flight altitude (from 100% at 2 m to 93.16% at 20 m); (2) yak dung density serves as a feasible proxy for GI, effectively capturing its temporal and spatial variability; (3) yak dung density reflects cumulative GI from May to September, and its representativeness increases with the length of accumulation. The proposed approach is characterized by high frequency, accuracy, and efficiency. It is well-suited for studying animal behavior and evaluating livestock–resource relationships, thereby providing valuable insights for sustainable grassland ecosystem management.

## Simple Summary

Grazing is the predominant utilization of grasslands worldwide. Nevertheless, accurately quantifying grazing intensity across spatial and temporal dimensions remains challenging. In this study, we introduced a novel approach to estimate grazing intensity by utilizing livestock dung as a proxy indicator in combination with repeated drone-based surveys. The feasibility of this method was demonstrated through a case study conducted in a household pasture on the Qinghai–Tibetan Plateau, China. The proposed approach demonstrates high frequency, accuracy, and operational efficiency, making it well suited for investigating grazing intensity and analyzing livestock-resource interactions.

## 1. Introduction

Grasslands, the most widespread and diverse ecosystems on Earth, are primarily used for livestock grazing worldwide [1]. Livestock provide diverse products and services, including meat, milk, hides, transportation and a source of income, capital and power [2]. In particular, livestock-derived meat and milk are essential for supporting several sustainable development goals and ensuring food security for millions of people worldwide [3,4]. Appropriate management supports the sustainable development of grassland structure and function, whereas poorly managed grazing can lead to environmental degradation, such as over-grazing [5]. Therefore, efficiently identifying grazing intensity and promptly adjusting grazing strategies has long been a central concern for researchers and grassland managers, particularly under climate change [6,7].

Traditionally, grazing intensity has been assessed through two main approaches: direct and indirect. The direct approach relies mainly on controlled-grazing experiments, which have been widely conducted in across grassland ecosystems [8,9], and have established the theoretical foundation for grass-livestock interactions. However, these experiments are costly, time-consuming, and labor-intensive. More importantly, considerable gap exists between controlled experimental settings and real-world grazing, limiting the practical applicability of the findings [10]. The indirect approach estimates grazing intensity through proxies derived from ground observations or equipment, e.g., observing the dung pats [11], livestock tracking with collars [12], and vegetation index (e.g., NDVI or EVI) monitoring with remote sensing data [13]. However, these methods often provide only rough approximations, which may not support precise or rational grassland management decisions. In recent years, UAVs have emerged as a novel remote sensing tool for monitoring animal populations and their spatial distribution [14,15]. Subsequent research has increasingly explored the application of UAVs in grazing management. For example, Luo et al. [16,17] developed an intelligent UAV system equipped with onboard deep learning algorithms for animal detection, tracking, and individual identification. In addition, researchers have developed automated counting algorithms to detect and enumerate animals in remote sensing images [18], and Groom et al. [19] outlined future directions for intelligent grazing management by proposing an integrated strategy combining UAV-based remote sensing, herd perception, and collaborative control. Nevertheless, quantifying grazing intensity in real-word situations remains difficult. Ji et al. [10] attempted to address this by using UAV images and kernel density estimation to quantify grazing intensity in household pastures. Despite these advances, quantifying grazing intensity in freely grazed areas at larger scales remains challenging.

Numerous studies have demonstrated a clear relationship between the temporal and spatial distribution of animal dung [20,21]. Especially, within a pasture, previous methods used to assess distribution and density of dung include manual mapping of dung across a pasture [22], transect establishment [23], use of the line intercept method [24], quadrat placement randomly or along transects [25,26]; or simply walking and marking dung pats and returning at a later date to monitor the changes [27]. In recent studies, some researchers documented that the morphological changes during the decomposition are relatively stable [28], and can be identified from UAV images [29,30]. Therefore, the relationship between livestock dung and grazing intensity offers a promising basis for precise quantification of grazing intensity at a larger scale. To date, few studies have quantified the relationships between grazing intensity and dung density.

The Qinghai–Tibetan Plateau (QTP) is referred to as the ‘third pole’, is a globally significant ecoregion [31]. Livestock grazing has been practiced on the QTP for thousands of years [32,33]. Yak (*Bos grunniens*) are the most important grazing livestock on the QTP, providing meat, milk and income for most residents of the region [32,34]. The Rangeland Contract Responsibility System (RCRS) was introduced to the QTP in the 1980s, after which household pastures became the basic management unit of alpine grassland [35]. In this study, we developed a UAV-based method to quantify the spatiotemporal distribution of grazing intensity and tested its feasibility in a household pasture on the QTP. The specific objectives were to (1) compare the effects of different flight altitudes on the accuracy of livestock dung pats identification; (2) establish a quantitative relationship between yak dung density and grazing intensity, and (3) assess how dung density reflects cumulative deposition over time. Widespread, long-term monitoring of grazing intensity using dung-based proxies, combined with high precision and frequent sampling, has the potential to improve understanding of livestock–resource interactions and contribute to better grassland ecosystem management.

## 2. Material and Method

### 2.1. Study Area

**Figure 1:**
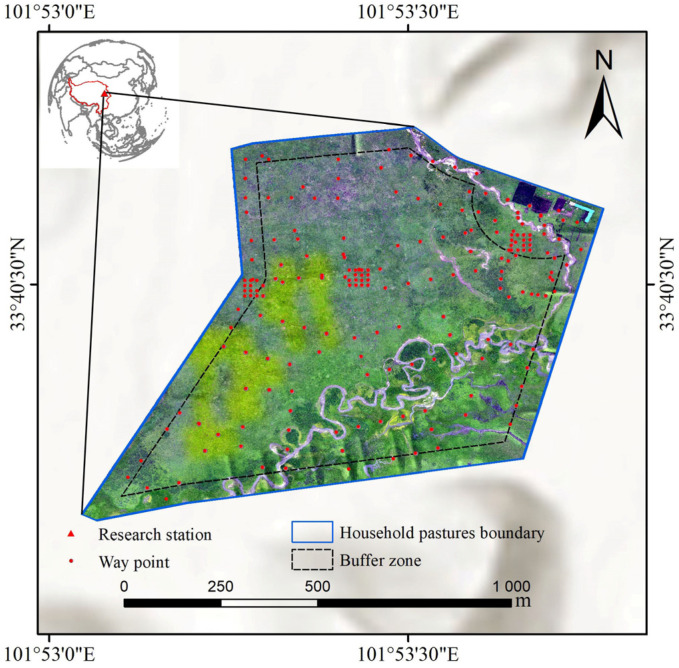
Study area in the east of the Qinghai–Tibetan Plateau.

This study was conducted from May to October in 2017, at Azi research station, Gansu Province, China (101°52′07.9″ E, 33°24′24.1″ N) (Figure 1). The study site has an average elevation of 3550 m, with mean annual precipitation >600 mm and mean annual air temperature of 1.1 °C [36]. The region has a typical plateau continental climate, characterized by cool, dry cold season and wet, humid warm season. The soil at the site is alpine meadow soil, and the vegetation is mainly alpine meadow dominated by monocotyledonous species, primarily Poaceae and Cyperaceae, along with diverse dicotyledonous species, e.g., *Ranunculaceae*, *Polygonaceae*, *Saxifragaceae*, *Asteraceae*, *Scrophulariaceae* and *Gentianaceae*.

A typical household pastures covering 113.64 ha with gentle topography (slope < 5°), were primarily used for warm-season grazing (Figure 1). A total of 278 yaks grazed in the pastures. Yaks were penned each night and released to graze during the daytime. Three to four neighboring night pens were used in rotation around each campsite (Figure 1). The pastures have been managed in this manner for more than 30 years. This practice was considered to generate a radial gradient of grazing intensity from the concentrator (i.e., the campsite) [10,37], providing a suitable field setting to study the relationship between yak dung density and grazing intensity, and to test the feasibility of the proposed method. To account for edge effects and the local herders’ collection of dung as fuel, a 50 m buffer zone was established along the fence as the boundary (Figure 1). Furthermore, a 150 m buffer zone was established around the livestock enclosure (the core area for dung collection and drying), and these areas were excluded from the study areas. This approach was designed to minimize the influence of unaccounted anthropogenic factors on the sample collection areas as much as possible (Figure 1).

### 2.2. Field Sampling and Data Collection

#### 2.2.1. Yak Dung Sampling Based on UAV

**Figure 2:**
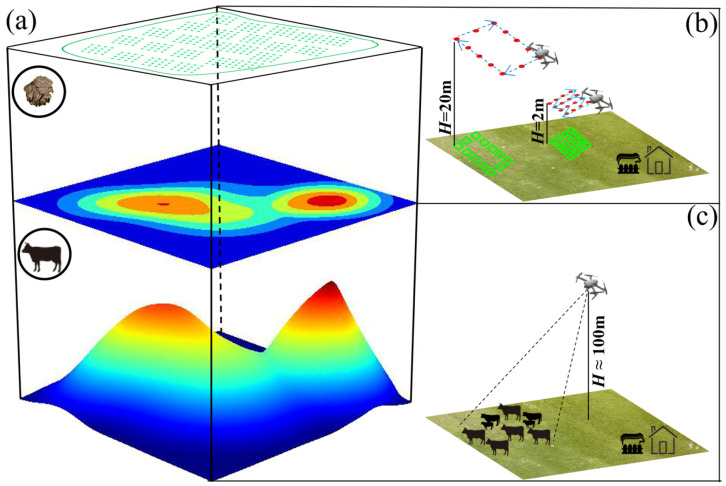
Field sampling methods for grazing intensity and yak dung density. (**a**) General framework of yak dung density and grazing intensity monitor; (**b**) the Belt and Rectangle routes of FragMAP used for monitoring the yak dung density in a typical household pasture (the red points represent waypoints, the blue dashed line indicates the UAV’s flight path, and the green rectangle shows the aerial imagery coverage area for either Rectangle or Belt route patterns); (**c**) dynamic monitoring of the spatiotemporal distribution of grazing intensity in typical household pastures.

From May to October, aerial images were collected monthly based on 12 Rectangle and 3 Belt routes using FragMAP, a self-developed APP based on the Software Development Kit (SDK) of DJI (Figure 2a) [38]. Some waypoints were excluded due to edge effects and herders’ fuel-collection activities. For the Rectangle route, the UAV (Phantom 3 Professional or Mavic Pro, DJI Innovation Company, Shenzhen, China, all the equipment sourced from China) was set on autopilot to fly over the 12 preset way points (100 m × 200 m) at a height of 20 m (Figure 2b). The UAV was equipped with a custom-designed camera (Phantom 3 Pro, Sensor: 1/2.3″ CMOS; Lens: FOV94°20 mm; Image Size: 4000 × 3000 pixels; Mavic Pro, Sensor: 1/2.3″ CMOS; Lens: 78.8°26 mm, Image Size: 3000 × 4000 pixels). Each aerial image covers roughly 35 m × 26 m (Phantom 3 Pro) and 29 m × 21 m (Mavic Pro), with a ground sampling resolution of roughly 1 cm^2^ per pixel [39]. For the Belt route, the Mavic Pro was used to autonomously fly over 16 preset way points (40 m × 40 m) at a height of 2 m. Each aerial image covers roughly 2.9 m × 2.1 m, with a ground sampling distance of ∼0.09 cm (Figure 2b) [36]. The ground coverage area of the aerial images was calculated using the following formula and validated through field verification.

$$\mathrm{Width}=\frac{S_{w}\times H}{f}÷1000\;Length=\frac{S_{l}\times H}{f}÷1000$$

where *H* is flight height (mm), *f* is focal length of the lens (mm), *S_l_* is length of the sensor (mm), S*_w_* is width of the sensor (mm), *Width* is ground coverage width (m) and *Length* is ground coverage length (m).

#### 2.2.2. Yak Dung Extraction on the Aerial Images

**Figure 3:**
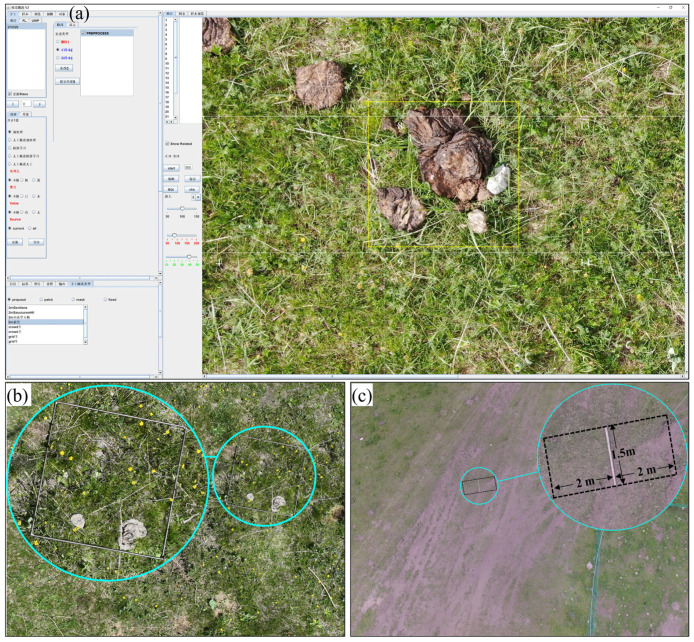
Yak dung identification and ground verification. (**a**) Yak dung identification based on aerial image using Object-rectV3 of FragMAP; (**b**,**c**) ground validation of Belt and Rectangle routes within areas of 0.25 m^2^ and 6 m^2^, respectively.

A Java-based software program (Object-rect.jar, version 3) within FragMAP was used to identify and count yak dung pats in the aerial images. Specifically, two trained researchers visually inspected each aerial image and delineated rectangles around the yak dung pats. In brief, the first researcher marks all the yak dung in each aerial image using Object-rectV3 (Figure 3a). The second researcher then corrected misidentified dung and added previously omitted pats to generate the final dataset. The marked samples were used for the subsequent training of the automated detection model.

#### 2.2.3. Samples Collection of Yak Dung for Ground Validation

During the aerial survey, some samples (i.e., aerial images) are randomly selected for ground sampling to validate the accuracy of the UAV-based sample collection. That is ground sampling was conducted monthly, a total of 30 samples were collected along the Rectangle routes (20 m height, 5 samples per month) from May to October, and 9 samples were obtained along the Belt routes (2 m height) in August. For accuracy validation, a 0.5 m × 0.5 m quadrat was used for the Belt routes (Figure 2b), while a 1.5 m reference stick delineating a 6 m^2^ area was used for Rectangle routes (Figure 2c). Field personnel counted all yak dung within these verification areas during the field surveys.

#### 2.2.4. Grazing Intensity Determination of Household Pasture

The method of grazing intensity determination in household pastures was described in detail in Sun et al. [40] and Ji et al. [10]. In brief, four to eight days of each month from May to October were randomly selected, and the hourly spatial distribution of the yak herd was monitored using UAV (Phantom 3 Pro), that is the UAV followed the herd and took an individual aerial image every hour during the day-time approximately at a height of 100 m. Aerial images taken with minimal disturbance to the herd can cover the entire herd by adjusting the shooting angle. There were 12–14 aerial images taken in one monitoring day, depending on the grazing schedule of the herder. During the monitoring period, two researchers and the herder conducted manual counts of the yak herd in the field to ensure data accuracy [40]. The locations of individual yaks were extracted through a process involving aerial image trimming, geometric correction, yak identification, and verification [10,40]. Finally, we used an adapted Kernel Density Estimation (KDE) approach to estimate grazing intensity. In this method, the integral of a single kernel function was redefined as the grazing intensity produced by one yak over one hour. The model incorporates pasture area, grazing duration, UAV sampling frequency, bandwidth, and an adjustment factor to compensate for the limited number of monitoring days. A triangular kernel with a bandwidth of 300 m—consistent with the hourly activity radius of yaks reported in prior studies—was applied [10]. Grazing intensity maps were produced at a 1 m spatial resolution (Figure S1). This study was conducted in the same region and time period as the previous research on yak grazing intensity, ensuring spatiotemporal comparability.

#### 2.2.5. Establish the Relationship Between Grazing Intensity and Dung Density

Grazing intensity data were extracted for all waypoints within the household pasture from the corresponding monthly grazing intensity maps, and the detail data from our previous study [10]. The potential relationship between grazing intensity and yak dung density was explored and quantified (Figure 2a).

### 2.3. Data Statistical Analysis

Normality and homogeneity of variances were first assessed using the Shapiro–Wilk test. If the assumptions of normality and homogeneity were met, ANOVA was conducted to determine significant differences in the effects of flight height on yak dung identification. The coefficient of determination (*R*^2^) and its *p*-values were used to assess the relationships between grazing intensity and yak dung density. All calculations and statistical analyses were performed using software R software version 4.3.1.

## 3. Results

### 3.1. The Accuracy of Identifying Yak Dung Based on Aerial Images

**Figure 4:**
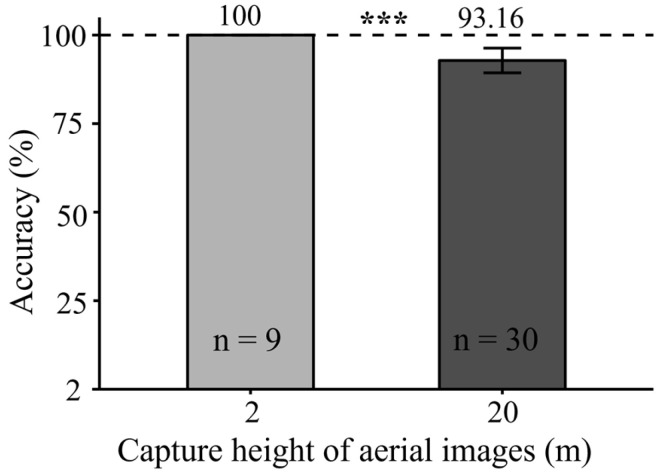
The accuracy of identifying yak dung pats based on aerial images derived from 2 m (*n* = 9) and 20 m (*n* = 30) flight height. *** represent the significance levels of *p* < 0.001.

The accuracy of yak dung identification in 2 m aerial images was significantly higher than that in 20 m aerial images (*p* < 0.001). Specifically, when the capture height was set at 2 m, all the yak dung was correctly identified, whereas accuracy dropped to approximately 93.16% at 20 m (Figure 4).

### 3.2. Relationships Between the Yak Dung Density and Grazing Intensity

**Figure 5:**
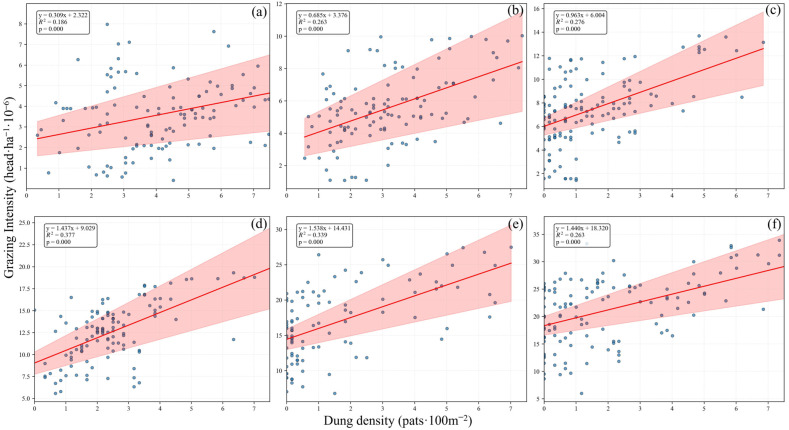
The relationships between the dung density and cumulative grazing intensity from May to October in a typical household pasture on eastern edge of the Qinghai–Tibetan Plateau. (**a**–**f**) represent the relationship between dung density and grazing intensity from May to October, respectively; the pink-shaded area represents the 95% confidence interval.

From May to October, the relationships between yak dung density and the cumulative grazing intensity were significantly positive (*p* < 0.001), and the correlation strengthened with increasing cumulative months, indicated by larger *R*^2^ values and smaller *p*-values. Furthermore, both the slopes and the intercepts of the fitting curves showed an increasing trend (Figure 5). Notably, commencing in July, yak dung density declined to zero at certain sampling sites. This decline corresponds to a period of reduced or absent dung pats from July onward, as shown in Figure 5 (pink-shaded area denotes the 95% confidence interval).

### 3.3. Suitability Variations in Yak Dung Density as a Proxy of Grazing Intensity

**Figure 6:**
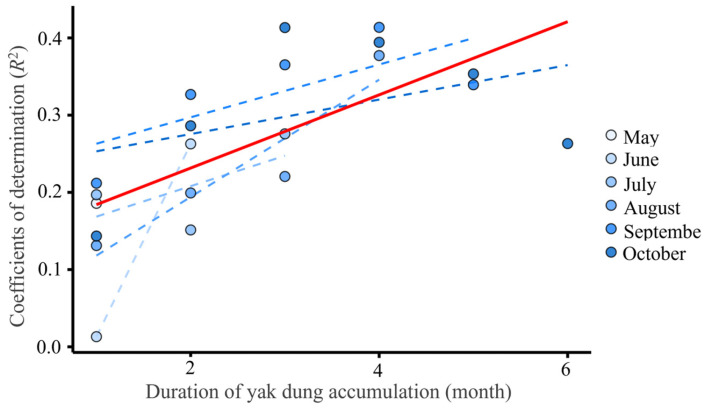
The variations in coefficients of determination (*R*^2^) of fitting curves between yak dung density and accumulated grazing intensities.

From May to October, the coefficients of determination (*R*^2^) increased with the duration of yak dung accumulation (*R*^2^ = 0.455, *p* < 0.01, Figure 6), indicated that the longer the livestock dung accumulates, the higher the accuracy in indicating grazing intensity within the same grazing season. In some months, the *R*^2^ were lower and the fitted curve weas not significant (*p* < 0.05), e.g., the fitted curve between yak dung density and grazing intensity in June (that is the grazing intensity was the estimated value of June, rather the cumulative grazing intensity from May) was not significant (Figure S2).

**Note:** The horizontal axis represents the duration of yak dung accumulation and associated grazing intensity. Numbers 1 to 6 correspond to different accumulation periods (in months), defined as follows: 1: Same-month pairing (i.e., May dung & May grazing, July dung & July grazing, August dung & August grazing, September dung & September grazing, and October dung & October grazing); 2: Dung density in a given month paired with grazing intensity accumulated over the previous two months (i.e., June dung & May–June grazing, July dung & June–July grazing, August dung & July–August grazing, September dung & August–September grazing, and October dung & September–October grazing); 3: Dung density paired with grazing intensity over the preceding three months (i.e., July dung & May–July grazing, August dung & June–August grazing, September dung & July–September grazing, October dung & August–October grazing); 4: Dung density paired with grazing intensity over the preceding four months (e.g., August dung & May–August grazing, September dung & June–September grazing, October dung & July–October grazing); 5: Dung density paired with grazing intensity over the preceding five months (i.e., September dung & May–September grazing, October dung & June–October grazing); 6: October dung density paired with full-season grazing intensity (May–October). Data source is Figure 5 and Figure S2.

## 4. Discussions

### 4.1. Quantification of the Relationship Between Yak Dung Density and Grazing Intensity

Grazing intensity serves as the key factor determining the effects of grazing on grasslands and underpins grazing management decisions and practices [9,30]. It has long been recognized that characteristics of livestock dung (e.g., number or weight) can indicate grazing density and distribution [4,29,41]. However, to date, most studies predominantly quantify dung density at varying distances from livestock water points or supplement feeding stations to validate its utility as an indicator of grazing intensity [29,41]. Although this approach can effectively assess spatial heterogeneity in relative grazing intensity at local scales, it faces methodological limitations in establishing quantifiable relationships between grazing intensity and dung density, therefore does not provide specific, measurable indicators of absolute grazing intensity.

It is widely recognized that the primary challenge lies in the practical difficulty of dynamically quantifying livestock grazing intensity under operational grazing management conditions. In recent years, Sun et al. [40] and Ji et al. [10] developed a methodology to quantify the spatiotemporal distribution of grazing intensity in household pasture systems. Building on this foundation, we established a quantitative relationship between grazing intensity and dung density at the household pasture scale using measured data (Figure 5). Furthermore, we explored the monthly relationships between dung density and grazing intensity, and the results showed that these relationships strengthened with increasing cumulative months. Therefore, this study not only provides a novel approach for quantifying livestock grazing intensity across multiple spatial scales, but also serves as an important reference for clarifying the regulatory mechanisms of livestock dung decomposition [42].

### 4.2. Identification of Livestock Dung

Accurate identification and analysis of livestock dung are critical steps for assessing grazing intensity. Traditional field sampling method can identify livestock dung correctly. However, sample areas and representativeness are usually limited. Moreover, these methods are temporally and spatially constrained and provide only a partial view of dung distribution dynamics and their interactions with vegetation communities, grazing patterns, and management strategies [18,25]. In recent years, UAVs have been used to monitor livestock dung dynamics due to their advantages of high spatial resolution, flexible timing for image acquisition, and cost efficiency [31,43]. Furthermore, support vector machine and YOLOv5 algorithm were used to detect livestock dungs, and demonstrated relatively high identification accuracy. However, the orthophoto-based monitoring approaches typically require considerable time and effort to acquire highly overlapping aerial images and perform image stitching [30]. Moreover, the large volume of images requires substantial storage capacity. This study adopts a long-term, fixed-point, collaborative monitoring strategy, which greatly enhances the efficiency of both field data collection and subsequent information extraction [44]. Furthermore, through the implementation of recoding and archiving methods, secure storage and precise retrieval of aerial images are achieved [38]. This establishes a comprehensive data management workflow encompassing data acquisition, information extraction, and secure preservation.

Beginning in July, dung density at certain sampling points declined to zero (Figure 5). Because a comprehensive ground survey corresponding to the aerial images was not conducted in this study, the exact reasons could not be determined. One possible reason is that overgrown vegetation obstructed visibility. Partial ground verification indicated that some yak dung was concealed by tall vegetation, while partially decomposed dung from the previous year and small bare soil patches were occasionally misidentified as livestock dung. Alternatively, dung absence may reflect livestock behavior, for instance, during the peak growing season, yaks can satisfy their nutritional requirements within limited areas, reducing the need to move frequently across the pasture [10]. Another potential reason could be the decomposition process of yak dung, which is highly variable depending on climate, vegetation and microenvironment [45]. For example, yak dung located in low-lying areas exhibits high moisture content and may even undergo runoff scour. Combined with active decomposition by microbes and soil fauna, it decomposes rapidly within a short period, thereby affecting the relationship between livestock dung density and grazing intensity. The observed increase in the slopes of the yak dung density-grazing intensity fitting curves could also be attributed to this factor (Figure 5).

### 4.3. Limitations and Future Prospects

Accurate quantification of livestock grazing intensity is essential for effective rangeland management and advancing related research. This study introduces a novel and comprehensive approach for assessing grazing intensity by integrating livestock dung indicators with UAV technology and the FragMAP system. This approach represents a substantial advancement, establishing a long-term, fixed-point, efficient, and coordinated monitoring and analysis framework that provides a foundation for developing smart pasture management systems. Nevertheless, further methodological refinements and technological enhancements are recommended for future research.

At the technical level: (1) repeated monitoring sometimes produced positional offsets. Although the UAV was programmed to follow the Rectangle and Belt routes of the FragMAP system, horizontal drift of approximately 1–2 m (Phantom 3 or Mavic Pro) occasionally occurred, potentially introducing errors into the surveyed area. This limitation was effectively resolved in subsequent studies through the integration of onboard RTK, which substantially improved the accuracy of monitoring and analysis. (2) the determination of an appropriate flight height for aerial images is also a critical issue. The accuracy of dung pats identification depends on both flight height and the resolution of the onboard camera [36]. At the same time, the area covered by each image (i.e., sample representativeness) must be considered comprehensively to determine a flight height that balances resolution with coverage, thereby ensuring accurate detection and analysis. (3) In this study, yak dung pats, were identified through visual interpretation, a process that was relatively inefficient and heavily dependent on the field experience and judgment of personnel. Nevertheless, the data extraction process using Object-rectV3 software generated a large number of standardized training samples, providing a solid foundation for the development of high-precision automated recognition models. Future research is expected to substantially enhance information extraction efficiency through machine learning models, thereby improving the timeliness and reliability of grazing intensity quantification.

At the theoretical and practical application levels: (1) some yak dung was located within river channel and could disappear rapidly during the rainy season (e.g., July and August) as rising water levels rose. In areas where riverbeds occupied nearly 50% of the aerial images’ extent, the number of visible dung pats decreased substantially, thereby biasing the relationship between dung density and grazing intensity. For this reason, such aerial images were excluded from the present analysis (Figure S3), and future studies should similarly exclude specific sampling points in order to improve the accuracy of the relationship between grazing intensity and dung density. (2) This study focused primarily on summer pastures within a relatively limited observation area. Future research should extend to larger spatial scales and examine the dung–grazing intensity relationship across multiple seasons, thereby quantifying these dynamics at regional or even broader scales. More broadly, research should aim to establish these relationships across diverse climatic and vegetation zones, facilitating the development of models applicable at broader spatiotemporal scales to guide livestock production practices. (3) Importantly, livestock dung density jointly determined by grazing intensity (i.e., livestock number and feeding frequency within a given area) and dung decomposition, the latter being a complex physicochemical process [45]. Future studies should place greater emphasis on elucidating the mechanisms of dung decomposition under varying biotic and abiotic conditions. Developing models that explicitly incorporate these processes will allow for more accurate simulation of the relationship between dung density and grazing intensity, thereby improving guidance for livestock production across larger spatial and temporal scales.

A dynamic dung decomposition model can quantify how decomposition biases the relationship between dung density and grazing intensity, enabling corrections of UAV-derived data. Moreover, future research could integrate decomposition processes into grazing intensity simulation models. This would enhance the accuracy of spatiotemporal predictions under real-world grazing conditions and provide more reliable decision support for regional grassland management.

## 5. Conclusions

The UAV-based monitoring approach developed in this study effectively characterizes the spatiotemporal distribution patterns of grazing intensity and expands the scope of investigation from local scales (e.g., individual household pastures) to entire regions with comparable environmental conditions. Nevertheless, several limitations should be addressed in the future studies, including determining the optimal UAV flight height and developing a high-accuracy automated detection model for livestock dung using artificial intelligence algorithms capable of distinguishing fresh dung from undecomposed dung of previous years. These improvements will ensure more efficient and precise identification of livestock dung. Additionally, research on livestock-grass interactions and the influence of biotic and abiotic factors will be highly valuable for the sustainable development of grassland ecosystems.

## Data Availability

The raw data supporting the conclusions of this article will be made available by the authors on request. Researchers interested in accessing the data should contact the corresponding authors.

## Supplementary Materials

The following supporting information can be downloaded at: https://www.mdpi.com/article/10.3390/ani15192789/s1, Figure S1: Distribution of grazing intensities in the study pasture from May to October [10]; Figure S2: The relationships between the dung density and cumulative grazing intensity of different months in a typical household pasture on eastern edge of the Qinghai-Tibetan Plateau; Figure S3: Some aerial images captured areas near rivers, where yak dung can be transported away during the wet season.
