# Supplementary material for: Livestock Dung Proxies Provide Insights into Grazing Density Quantification and Distribution
Source: Animals (Basel). 2025 Sep 25;15(19):2789. doi: 10.3390/ani15192789 (PMC12523358; doi:10.3390/ani15192789)
Supplement: Supplementary file 1 [file animals-15-02789-s001.zip › animals-3870925-supplementary.pdf]

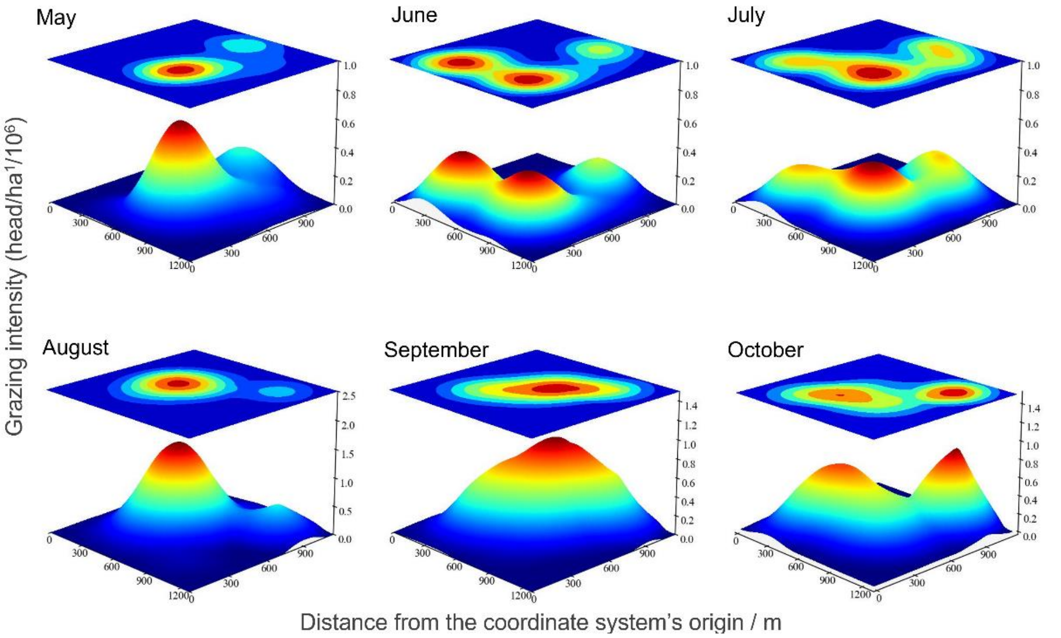

Figure S1. Distribution of grazing intensities in the study pasture from May to October [10].

10. Ji, W.; Luo, Y.; Liao, Y.; Wu, W.; Wei, X.; Yang, Y.; He, X.Z.; Shen, Y.; Ma, Q.; Yi, S.; Sun, Y. UAV Assisted Livestock Distribution Monitoring and Quantification: A Low-Cost and High-Precision Solution. *Animals* **2023**, *13*, 3069.

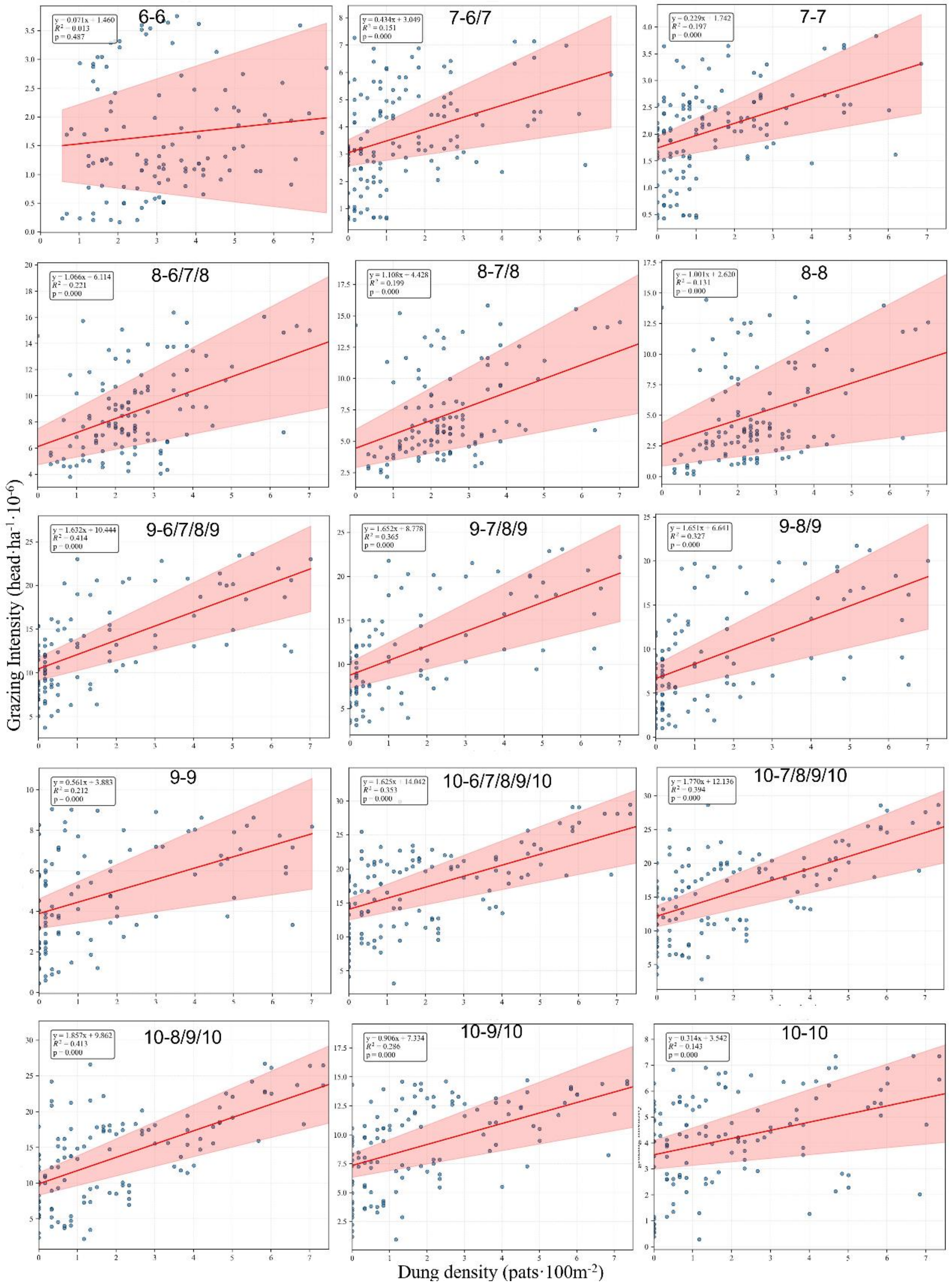

Figure S2. The relationships between the dung density and cumulative grazing intensity of different months in a typical household pasture on eastern edge of the Qinghai-Tibetan Plateau.

The numbers on each figure indicate the yak dung sampling time (left) and duration of cumulative grazing intensity (right), respectively.

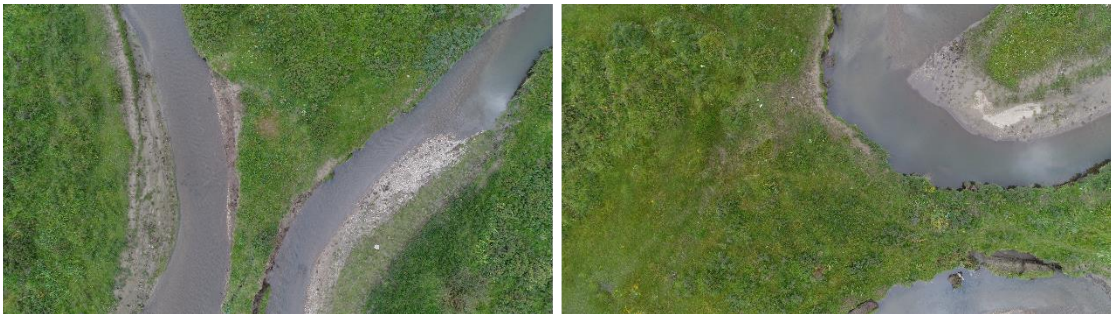

Figure S3. Some aerial images captured areas near rivers, where yak dung can be transported away during the wet season
